# Effects of high doses of zearalenone on some antioxidant enzymes and locomotion of *Tenebrio molitor* larvae (Coleoptera: Tenebrionidae)

**DOI:** 10.1093/jisesa/ieae052

**Published:** 2024-05-08

**Authors:** Milena Janković-Tomanić, Branka Petković, Jelena S Vranković, Vesna Perić-Mataruga

**Affiliations:** Department of Insect Physiology and Biochemistry, Institute for Biological Research “Siniša Stanković”—National Institute of the Republic of Serbia, University of Belgrade, Despot Stefan Blvd. 142, 11108 Belgrade, Serbia; Department of Neurophysiology, Institute for Biological Research “Siniša Stanković”—National Institute of the Republic of Serbia, University of Belgrade, Despot Stefan Blvd. 142, 11108 Belgrade, Serbia; Department of Hydroecology and Water Protection, Institute for Biological Research “Siniša Stanković”—National Institute of the Republic of Serbia, University of Belgrade, Despot Stefan Blvd. 142, 11108 Belgrade, Serbia; Department of Insect Physiology and Biochemistry, Institute for Biological Research “Siniša Stanković”—National Institute of the Republic of Serbia, University of Belgrade, Despot Stefan Blvd. 142, 11108 Belgrade, Serbia

**Keywords:** mealworm, mycotoxin, superoxide dismutase, glutathione S-transferase, locomotion

## Abstract

The mealworm *Tenebrio molitor* L. (Coleoptera: Tenebrionidae) feeds on wheat bran and is considered both a pest and an edible insect. Its larvae contain proteins and essential amino acids, fats, and minerals, making them suitable for animal and human consumption. Zearalenone (ZEA) is the mycotoxin most commonly associated with *Fusarium spp.* It is found in cereals and cereal products, so their consumption is a major risk for mycotoxin contamination. One of the most important effects of ZEA is the induction of oxidative stress, which leads to physiological and behavioral changes. This study deals with the effects of high doses of ZEA (10 and 20 mg/kg) on survival, molting, growth, weight gain, activity of antioxidant enzymes superoxide dismutase (SOD) and glutathione S-transferase (GST), and locomotion of mealworm larvae. Both doses of ZEA were found to (i) have no effect on survival, (ii) increase molting frequency, SOD, and GST activity, and (iii) decrease body weight and locomotion, with more pronounced changes at 20 mg/kg. These results indicated the susceptibility of *T. molitor* larvae to high doses of ZEA in feed.

## Introduction

From a human perspective, insects such as mealworms *Tenebrio molitor* L. (Coleoptera: Tenebrionidae) are generally considered pests in agroecosystems, because they eat stored grains and grain products worldwide (Rosentrater 2022). On the other hand, mealworms are used as livestock feed, because of their nutritious properties—proteins, fats, and minerals (van Raamsdonk et al. 2017, Hong et al. 2020, Lee et al. 2022, Syahrulawal et al. 2023). Various bioactive peptides extracted from the mealworm have antioxidant and antimicrobial properties (Kim et al. 2023). Mealworm larvae can be successfully reared on agricultural waste and by-products of the brewing and baking industries (Mancini et al. 2019), which carry the risk of contamination with mycotoxins (van der Fels-Klerx et al. 2018). Most available studies on insects in relation to mycotoxin exposure focus on accumulation/retention and safety aspects of their use as food supplements (Ochoa Sanabria et al. 2019, Niermans et al. 2021). Mycotoxins can potentially affect molting and pupation in insects (Azab et al. 2001).

Zearalenone (ZEA) is a ubiquitous and widely distributed mycotoxin (Ropejko and Twarużek 2021) with a range of adverse effects on animals and humans. One of the most important aspects of ZEA toxicity is oxidative stress (Hann et al. 2022, Llorens et al. 2022), as a result of an imbalance between reactive oxygen species (ROS) generated by mycotoxins and the antioxidant capacity of cells (da Silva et al. 2018), which contributes to physiological and behavioral changes (Kumsta et al. 2011). At low levels of contamination, as well as during short exposure routes, mycotoxins can induce antioxidant mechanisms and alter enzymes with antioxidant and/or detoxifying activities. In contrast, longer routes of contamination, or high levels of mycotoxins, can reduce antioxidant defenses, and suppress the expression and activity of antioxidant enzymes (Mavrommatis et al. 2021, Feng et al. 2022). First-line antioxidant enzymes such as superoxide dismutase (SOD) and catalase, followed by ascorbate peroxidase and glutathione reductase, which are present in most insect species, provide an efficient system to cope with oxygen free radicals. Glutathione S-transferase (GST) could actively participate in an insect’s antioxidant defense, due to its role as a Se-independent peroxidase in insects (Simmons et al. 1989).

The aim of this study was to investigate the effects of high doses of ZEA (10 and 20 mg/kg feed) on survival, growth, molting, pupation, weight gain, activity of SOD and GST, and locomotion of mealworm larvae. ZEA concentrations usually range from 0.015 to 5.7 mg/kg (Schollenberger et al. 2006), but the occurrence of ZEA in individual samples can even reach 14.6 mg/kg depending on the season (Streit et al. 2012).

## Materials and Methods

*Tenebrio molitor* larvae weighing between 100 and 120 mg, which corresponded to the 12^th^/13^th^ larval instar, were fed ad libitum with pure wheat bran (‘Kikindski mlin’, Kikinda, Serbia) with the addition of 0.5% dry yeast (‘Centroproizvod’, Belgrade, Serbia) (control). Larvae were reared under controlled conditions (24 ± 1°C, 60 ± 10% relative humidity, 100 lux light intensity, 12 h light:12 h dark. Body weight was measured before the start of the experiment and after the first and second week. The experimental groups were treated for 2 weeks with ZEA (CAS number 17924-92-4, Sigma-Aldrich, St. Louis, MO, USA), dissolved in ethanol (10 ml spiking solution/20 g wheat bran/20 larvae/3 replicates per group), at a concentration of 10 and 20 mg/kg (ZEA 10 and ZEA 20, respectively). The substrates were spiked at a concentration of either 20 × the maximum levels (ML), and approx. 7 × guidance values (GV), in relation to the levels relative to EC MLs and GVs (EC 2006), respectively.

For the analysis of survival, weight gain, molting and pupation, oxidative stress parameters, locomotion, and mycotoxin content in the larvae and residues (according to Janković-Tomanić et al. 2023) larval samples were randomly collected from each replicate. It is encouraging that the values of the analyzed parameters did not vary greatly within the experimental groups, so despite the limited number of larvae, the effect of the mycotoxin is clearly noticeable.

Mycotoxin content was assessed by high-performance liquid chromatography with a quantification limit of 5 µg/kg (Belgrade City Institute for Public Health).

To determine enzyme activity larvae were homogenized in 0.25 M sucrose buffer (pH 7, 100 mg/2 ml) for 3 × 10 s at 2,000 rpm using an Ultra Turrax homogenizer (IKAWerke, Staufen, Germany) followed by 3 × 15 s steps in a 50-W sonifier (Bandelin sonopuls HD2070, Berlin, Germany). Sonicates were centrifuged at 105,000 × g at 4 °C (Beckman, L7-55, Ultracentrifuge, Nyon, Switzerland), and supernatants were decanted and frozen at −80 °C until use. SOD activity was measured spectrophotometrically at λ = 480 nm, as described by Misra and Fridovich (1972), and expressed as the amount of enzyme causing 50% inhibition of epinephrine autooxidation, in units per milligram of protein. GST was measured spectrophotometrically at λ = 340 nm by the method of Habig et al. (1974) and expressed in nanomoles of glutathione (GSH) per minute per milligram of protein. Protein concentration was determined by the method of Bradford (1976), using bovine serum albumin as a standard. Spectrophotometric measurements were performed using a Shimadzu UV-1800 spectrophotometer (USA).

Locomotion was recorded using a webcam (Logitech Inc., Fremont, CA, USA) for 5 min under the same rearing conditions, analyzed using Any-maze software (version 6.35, Stoelting, Wood Dale, USA), and expressed as travel distance (m), time in movement (s), and average speed while in motion (m/s).

The Kolmogorov–Smirnov test was used for testing the normality of data. Parametric tests repeated measures analysis of variance (ANOVA) and 1-way ANOVA, followed by appropriate post hoc tests, were used to evaluate the effect of ZEA on body weight and antioxidant enzyme activity, respectively. Non-parametric Kruskal–Wallis test, followed by an appropriate post hoc test, was used to evaluate the effect of ZEA on locomotion.

## Results

Mealworm larvae fed ZEA-contaminated feed showed no significant changes in survival, but the presence of the mycotoxin significantly affected larval molting and pupation, which occurred more frequently in the treated groups (37.5% in ZEA 10 and 48.3% in ZEA 20) than in the control group (3.9%).

At a concentration of 10 mg/kg ZEA in the feed, the mycotoxin content in the residues was 5.154 mg/kg (i.e., 51.54%), while in the larvae it was 0.032 mg/kg (i.e., 3.2%). The ZEA content in the residues of the ZEA 20 group was almost 19 mg/kg (i.e., 95%) of the mycotoxin in the feed, while the larvae retained 0.113 mg/kg (i.e., 11.3%) in their bodies.

The presence of high doses of ZEA in feed significantly affected the body weight of mealworm larvae (treatment: *F*_(2,84)_ = 152.1, *P* = 0.001, week: *F*_(2,168)_ = 65.4, *P* = 0.001, and treatment × week: *F*_(4,168)_ = 56.6, *P* = 0.001). In the control group, larval body weight increased significantly over time. In the ZEA 10 and especially the ZEA 20 group, larval body weight decreased significantly after the first week and then increased, but statistically significantly only in the ZEA 10 group after the second week of treatment (Table 1).

**Table 1.** Temporal changes in body weight of mealworm larvae reared on pure wheat bran (control) or contaminated with ZEA at a concentration of 10 and 20 mg/kg (ZEA 10 and ZEA 20, respectively). Results are presented as mean ± SEM (*n* = 29 larvae per group). Different letters next to values indicate significant differences within columns (LSD test)

	Larval body weight (mg)		
	Control	ZEA 10	ZEA 20
Initial	110.5 ± 0.5 a	113.3 ± 0.5 a	113.4 ± 0.5 a
1^st^ week	122.7 ± 1.8 b	109.4 ± 0.7 b	107.6 ± 0.6 b
2^nd^ week	139.9 ± 1.6 c	116.8 ± 1.3 c	109.8 ± 1.6 b

**Fig. 1.**
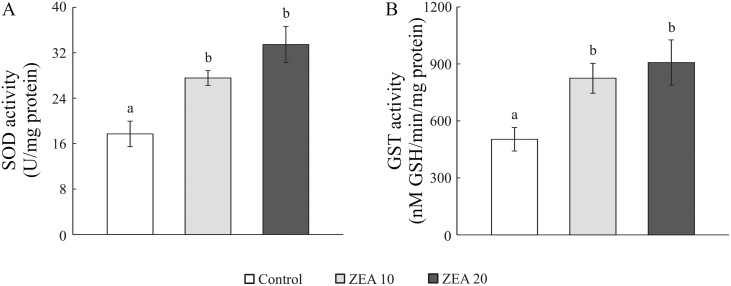
The activity of A) superoxide dismutase (SOD) and B) glutathione S-transferase (GST) in mealworm larvae reared on pure wheat bran (control) or contaminated with ZEA at a concentration of 10 and 20 mg/kg (ZEA 10 and ZEA 20, respectively). Each bar represents the mean ± SEM (*n* = 10 larvae per group). Different letters above the bars indicate significant differences between groups (LSD test).

The activity of SOD and GST was altered by the presence of ZEA in feed (*F*_(2,27)_ = 11.3, *P* = 0.001 and *F*_(2,27)_ = 5.7, *P* = 0.009, respectively). This was reflected in a significant increase in the activity of both enzymes in the ZEA 10 and ZEA 20 groups compared to the control group (Fig. 1). The increase was particularly marked in the ZEA 20 group but without a significant difference compared to the ZEA 10 group.

**Fig. 2.**
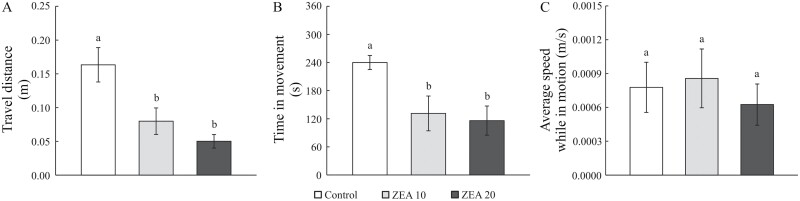
Locomotor activity expressed as A) travel distance, B) time in movement, and C) average speed while in motion in mealworm larvae reared on pure wheat bran (control) or contaminated with ZEA at a concentration of 10 and 20 mg/kg (ZEA 10 and ZEA 20, respectively). Each bar represents the mean ± SEM (*n* = 7–9 larvae per group). Different letters above the bars indicate significant differences between groups (Mann–Whitney *U* test).

The presence of ZEA in feed affected the locomotion of mealworm larvae, namely travel distance (*H*_(2,24)_ = 12.7, *P* = 0.002) and time in movement (*H*_(2,24)_ = 8.8, *P* = 0.012), whereas average speed while in motion was unchanged (*H*_(2,24)_ = 0.5, *P* = 0.8). Compared with the control group, significantly decreased travel distance and time in movement were observed in the ZEA 10 group and to an even greater extent in the ZEA 20 group (Fig. 2). Although the values of the analyzed parameters were lower in the ZEA 20 group, they were not significantly different from those of the ZEA 10 group.

## Discussion

Our results indicate that mealworm larvae are able to survive on ZEA-contaminated feed at such high concentrations (10 and 20 mg/kg). Mortality rates of mealworm larvae exposed to various mycotoxins ranged from 2%, 7%, and 11%, and susceptibility to mycotoxins appears to be stage- and dose-dependent (Van Broekhoven et al. 2017, Camenzuli et al. 2018, Ochoa-Sanabria et al. 2019, Piacenza et al. 2021). The high survival rate of mealworm larvae reflects the fact that the effects of mycotoxins are less harmful in the later larval stages, which was the case in our experiment (Janković-Tomanić et al. 2023). In general, insects show little or no mortality in response to mycotoxins (Abado-Becognee et al. 1998, Bosch et al. 2017), especially insects of the order Coleoptera compared to, for example, Lepidoptera and Diptera (Niermans et al. 2021, 2023).

Regarding the presence of mycotoxins in residues, ZEA accounts for 50% and even 95% of mycotoxins in feed. It is possible that larvae are unable to metabolize mycotoxins at such high concentrations. There is no evidence that mycotoxins accumulate in the tissue of insect larvae, with the Coleoptera species having a high excretory capacity (Bisconsin-Junior et al. 2023). However, ZEA impaired molting, which occurred more frequently. This may be due, at least in part, to the action of the mycotoxin as an endocrine disruptor (Demaegdt et al. 2016). Although insects do not have a ZEA-specific target site, the increased frequency of molting and pupation could be due to effects on the function of the hormone ecdysone, which controls development and molting in insects.

Mealworms exposed to high concentrations of ZEA (10 and 20 mg/kg) showed weight loss, which was especially evident after the first week of treatment. In general, mycotoxin exposure can affect digestion, absorption, and metabolism of nutrients (Jia et al. 2020, Peillod et al. 2021, Xu et al. 2022). Specifically, ZEA can lead to decreased feed intake, feed conversion efficiency, utilization of digested food, and/or damage the gastrointestinal tract (Vasatkova et al. 2009, Furian et al. 2022), resulting in weight loss. The disappearance of the effect at a dose of 10 mg/kg after the second week of treatment suggests that larvae have mechanisms to overcome the toxic effects of ZEA that appear to be dose-dependent.

It is known that ZEA is able to generate free radicals in a dose-dependent manner and alter the expression and activity of enzymes and genes associated with oxidative stress (Vidal et al. 2018, Llorens et al. 2022). Obtained results showed that ZEA at concentrations of 10 and 20 mg/kg induced a change in the activity of SOD and GST, with a tendency to increase at higher doses of ZEA. This could be a mechanism to destroy excess ROS caused by ZEA (Huang et al. 2021, Feng et al. 2022), and an indication that the enzymes reach their maximum capacity at about 10 mg/kg, so further increasing mycotoxin concentration cannot increase antioxidant protection. It can be concluded that antioxidant enzymes are modulated differently depending on the load caused by mycotoxins (Yoon et al. 2019, Mavrommatis et al. 2021).

In the present study, ZEA affected the locomotion of larvae by decreasing travel distance and time in movement, with no changing average speed while in motion. This is consistent with previous findings suggesting that some mycotoxins cause behavioral changes associated with their neurotoxic effects (Baldissera et al. 2018, Janković-Tomanić et al. 2019, Huang et al.,2021) and could be explained by the influence of ZEA on the central neuronal elements involved in the control of locomotion (Mantziaris et al. 2020).

Our results suggest that ZEA at concentrations of 10 and 20 mg/kg is capable of inducing biochemical, physiological, and behavioral changes in *T. molitor* larvae that could be considered early markers of mycotoxin intoxication. Given the observed increased activity of antioxidant enzymes, it is reasonable to assume that induction of oxidative stress, as one of the main aspects of mycotoxin toxicity, is the first event that triggers all the others, leading to increased frequency of molting and pupation, loss of body weight, and decreased locomotion.
